# Influence of preterm birth on the association between gestational diabetes mellitus and childhood developmental vulnerability: a causal mediation analysis

**DOI:** 10.1007/s12519-023-00741-7

**Published:** 2023-07-31

**Authors:** Bereket Duko, Amanuel Tesfay Gebremedhin, Gizachew Assefa Tessema, Gavin Pereira

**Affiliations:** 1 Australian Centre for Precision Health, UniSA Clinical and Health Sciences, University of South Australia, North Terrace, Adelaide, SA 5000 Australia; 2 Curtin School of Population Health, Faculty of Health Sciences, Curtin University, Kent Street, Bentley, WA 6102 Australia; 3 enAble Institute, Curtin University, Kent Street, Bentley, WA 6102 Australia; 4 Centre for Fertility and Health (CeFH), Norwegian Institute of Public Health, Oslo, Norway

**Keywords:** Child development, Causal mediation analysis, Gestational diabetes mellitus, Offspring

## Abstract

**Background:**

Epidemiological studies examining the direct and indirect effects of gestational diabetes mellitus (GDM) on offspring early childhood developmental vulnerability are lacking. Therefore, the aims of this study were to estimate the direct and indirect effects of GDM (through preterm birth) on early childhood developmental vulnerability.

**Methods:**

We conducted a retrospective population-based cohort study on the association between gestational diabetes mellitus and early childhood developmental vulnerability in children born in Western Australia (WA) using maternal, infant and birth records from the Midwives Notification, Hospitalizations, Developmental Anomalies, and the Australian Early Development Census (AEDC) databases. We used two aggregated outcome measures: developmentally vulnerable on at least one AEDC domain (DV1) and developmentally vulnerable on at least two AEDC domains (DV2). Causal mediation analysis was applied to estimate the natural direct (NDE), indirect (NIE), and total (TE) effects as relative risks (RR).

**Results:**

In the whole cohort (*n* = 64,356), approximately 22% were classified as DV1 and 11% as DV2 on AEDC domains. Estimates of the natural direct effect suggested that children exposed to GDM were more likely to be classified as DV1 (RR = 1.20, 95% CI: 1.10–1.31) and DV2 (RR = 1.34, 95% CI: 1.19–1.50) after adjusting for potential confounders. About 6% and 4% of the effect of GDM on early childhood developmental vulnerability was mediated by preterm birth for DV1 and DV2, respectively.

**Conclusion:**

Children exposed to gestational diabetes mellitus were more likely to be developmentally vulnerable in one or more AEDC domains. The biological mechanism for these associations is not well explained by mediation through preterm birth.

**Supplementary Information:**

The online version contains supplementary material available at 10.1007/s12519-023-00741-7.

## Introduction

Early childhood development, prenatal to age five years, is considered the most sensitive period in human life, as the brain develops more rapidly than at any other time in life [1]. Such development encompasses several neurological and psychomotor skills [2], including physical health and well-being, social and emotional, cognitive, and language capacities of the child in the first five years of life [3]. Unhealthy childhood development may have long-term negative consequences for children’s future overall physical health and well-being, behavior, social adjustment, skill acquisition, and academic achievement [3, 4]. Several direct and indirect experiences during the prenatal and postnatal periods are widely believed to compromise early childhood development [5–7]. Therefore, it is imperative to thoroughly examine such early life putative factors of childhood developmental vulnerability to improve unfavorable adverse outcomes later in life.

Gestational diabetes mellitus is one of the most common metabolic disturbances occurring during pregnancy, complicating about 10% of pregnancies globally [8], which in turn may result in adverse physical, neuropsychological, and psychological sequelae in their children. Epidemiological evidence suggests that GDM is associated with lower scores of mental and psychomotor development at ages 1–2 years [9], gross and fine motor abnormalities [10, 11], higher rates of attention deficit and lower cognitive scores in children younger than 9 years [11], poorer language development [12, 13], lower intelligence quotient (IQ) and impoverished behavioral and emotional functioning [14]. Nonetheless, additional studies concluded that there is insufficient statistical evidence to claim such an association [15–17]. Conflicting results may be due to variability in the selection of potential confounders and lack of consideration of mediation by preterm birth.

Being born preterm may contribute to several adverse effects on future health and well-being, including early childhood developmental vulnerability. For instance, children of mothers with GDM are at increased risk of being born preterm [18], which in turn could explain the effects of GDM on early childhood developmental vulnerability at school entry [19]. Moreover, studies examining whether GDM is associated directly with early childhood developmental vulnerability or indirectly through preterm birth are lacking. The aims of this study were to estimate the natural direct, indirect, and total effects of GDM on childhood developmental vulnerability in Australian Early Development Census (AEDC) domains and the proportion of the effect of GDM on early childhood developmental vulnerability in AEDC domains mediated by preterm birth in children born in Western Australia (WA).

## Methods

### Study design and data sources

We conducted a retrospective population-based cohort study on the association between gestational diabetes mellitus and early childhood developmental vulnerability in AEDC domains in children born in Western Australia using maternal, infant and birth records from the Midwives Notification System (MNS), a statutory data collection of all births (live or still born) in WA of at least 20 weeks gestation and/or birthweight greater than 400 g if the gestational length was unknown [20]. The MNS records were cross-validated with corresponding records from the WA birth registry [21]. Data on developmental anomalies, which were collected for all diagnoses up until the age of six years, were also obtained from the WA Register for Developmental Anomalies (WARDA). Hospitalization data were also sourced from the WA Hospital Morbidity Data Collection (HMDC), which includes almost all hospitalizations in WA [22]. Data on early childhood developmental vulnerability in AEDC domains for children born in WA were obtained from the AEDC 2009-, 2012- and 2015-year records from the Australian Department of Education.

### Inclusion and exclusion criteria

This study cohort consisted of 71,654 singleton children who were born in WA and were included in the 2009, 2012, and 2015 assessment waves of the AEDC. We sequentially excluded children who were identified as having “special needs” based on a diagnosed physical and/or intellectual disability (*n* = 2723), had a congenital anomaly (*n* = 3449), had incomplete AEDC scores (*n* = 887), and had missing data on gestational diabetes mellitus (*n* = 239). These exclusions resulted in a cohort of 64,356 mother–child pairs.

### Exposure: gestational diabetes mellitus

Gestational diabetes mellitus was ascertained from the Midwives Notifications System (yes/no) and the HMDC separately. HMDC uses the International Classification of Diseases (ICD-9/10) diagnostic codes consistent with gestational diabetes mellitus (ICD-9-AM: 648.8, ICD-10-AM: O24.4). According to the Australasian Diabetes in Pregnancy Society (ADIPS) guidelines, a diagnosis of GDM can be made if one or more of the following glucose levels are elevated: fasting plasma glucose > 5.1 mmol/L, 1-hour post 75 g oral glucose load > 10.0 mmol/L and 2-hour post 75 g oral glucose load > 8.5 mmol/L [23].

### Mediator: preterm birth

Data on preterm birth were sourced from the MNS. Preterm birth (yes/no) was defined as live birth prior to 37 completed weeks of gestation.

### Outcome: early childhood developmental vulnerability in AEDC domains

Data on early childhood developmental vulnerability in AEDC domains at the median age of five years were obtained from the AEDC records. The AEDC is a population-based census adapted from the Canadian Early Development Instrument to measure five domains of early childhood developmental vulnerability: physical health and well-being, social competence, emotional maturity, language and cognitive skills, and communication skills and general knowledge [24]. According to the AEDC, the physical health and well-being domain measures children’s physical readiness for the school day, physical independence, and gross and fine motor skills. The emotional maturity domain measures children’s pro-social and helping behavior, anxious and fearful behavior, aggressive behavior and hyperactivity, and inattention behaviors. The language and cognitive skills (school based) domain measures children’s basic literacy, interest in literacy, numeracy and memory, advanced literacy, and basic numeracy. The social competence domain captures children’s overall social competence, approaches to learning and readiness to explore new things, and responsibility and respect. The communication skills and general knowledge domain measures children’s communication skills and general knowledge. These five areas of childhood developmental vulnerability in AEDC domains are closely associated with predictors of good health, social outcomes, and education later in life [25]. The AEDC is completed by a teacher when children enter their first year of full-time school and is conducted in Australia every three years [26]. The AEDC was first conducted nationally in 2009. The AEDC cutoff scores are based on the 2009 data collection and apply to all subsequent data collections. Following the 2009 data collection, children with scores below the 10th percentile in a given domain are classified as “developmentally vulnerable” [27]. Domain scores can be calculated for all children, but those students classified as “special needs” are excepted, as the AEDC was not validated for this population group. For this study purpose, we used two aggregated outcome measures: developmentally vulnerable on one or more AEDC domains (DV1) and developmentally vulnerable on two or more AEDC domains (DV2).

### Covariables and confounders

We selected a range of maternal, child and family level characteristics based on results from previous epidemiological studies and their availability in our datasets (Table 1). Sociodemographic risk factors including maternal age (< 20, 20–24, 25–29, 30–34, 35–39 and ≥ 40 years), marital status (married, de facto, unmarried, divorced, separated), ethnicity (Caucasian, Indigenous Australians, all others) and parity (nulliparous, primiparous, multiparous), maternal tobacco smoking status during pregnancy (yes/no), and index of relative sociodemographic disadvantage were available in the MNS. Previous epidemiological studies have suggested that cesarean-born children, when compared to vaginally born children, are at increased risk of being developmentally vulnerable across multiple domains [28]. Data on cesarean section delivery (yes/no) were also available on the MNS. The index of relative sociodemographic disadvantage (IRSD) was calculated using the residential address at the time of childbirth and categorized into quintiles (< 20th—most disadvantaged, 20 to 39th, 40 to 59th, 60 to 79th and ≥ 80th—least disadvantaged) that reflect area-level sociodemographic disadvantage considering factors such as low educational attainment, low household income, and high levels of unemployment [29]. This IRSD index was obtained from the Australian Bureau of Statistics (ABS). Child variables including child’s age at the time of AEDC completion (≥ 4 years to 5 years and 1 month/ ≥ 5 years and 1 month to 5 years and 10 months/ ≥ 5 years and 10 months), language other than English spoken at home (yes/no), child’s indigenous status, and child’s place of birth (Australia/any other country) were obtained from the AEDC records. We have previously found that preeclampsia and low birthweight are associated with early childhood developmental vulnerability in AEDC domains, but these variables were not included in the analysis, as we assumed that hypertensive disorders of pregnancy and fetal growth restriction are not direct biological causes of GDM.

**Table 1:** Maternal and child characteristics by exposure status (*n* = 64,356)

Maternal and child characteristics	Total	Gestational diabetes mellitus	
		No (*n* = 61,297)	Yes (*n* = 3059)
	*n* (%)	*n* (%)	*n* (%)
Maternal age at childbirth, y			
< 20	4682 (7.3)	4604 (7.5)	78 (2.6)
20–24	8537 (13.3)	8321 (13.6)	216 (7.1)
25–29	17,647 (27.4)	16,945 (27.6)	702 (23.0)
30–34	20,599 (32.0)	19,545 (31.9)	1054 (34.5)
35–39	10,814 (16.8)	10,020 (16.4)	794 (26.0)
≥ 40	2077 (3.2)	1862 (3.0)	215 (7.0)
Ethnicity			
Caucasian	53,157 (82.6)	51,001 (83.2)	2156 (70.5)
Indigenous Australian	3535 (5.5)	3338 (5.5)	197 (6.4)
All other	7664 (11.9)	6958 (11.3)	706 (23.1)
Index of Relative Socioeconomic Disadvantage (quintiles)			
Less than 20th	12,647 (20.0)	12,028 (20.0)	619 (20.6)
20 to 39th	12,607 (20.0)	11,965 (19.9)	642 (21.3)
40 to 59th	12,581 (20.0)	11,970 (20.0)	611 (20.3)
60 to 79th	12,593 (20.0)	11,983 (20.0)	610 (20.3)
Greater than or equal to 80th	12,576 (20.0)	12,049 (20.1)	527 (17.5)
Marital status			
Married	57,703 (89.7)	54,911 (89.6)	2792 (91.3)
All other	6178 (9.6)	5928 (9.7)	250 (8.2)
Missing	475 (0.7)	458 (0.8)	17 (0.6)
Parity			
Nulliparous	26,459 (41.1)	25,227 (41.2)	1232 (40.3)
Primiparous	22,178 (34.5)	21,201 (34.6)	977 (31.9)
Multiparous	15,719 (24.4)	14,869 (24.3)	850 (27.8)
Maternal prenatal tobacco smoking			
No	54,247 (84.3)	51,546 (84.1)	2701 (88.3)
Yes	10,109 (15.7)	9751 (15.9)	358 (11.7)
Preterm birth			
No	60,242 (93.6)	57,456 (93.7)	2786 (91.1)
Yes	4110 (6.4)	3837 (6.3)	273 (8.9)
Cesarean section delivery			
No	43,604 (67.8)	41,850 (68.3)	1754 (57.3)
Yes	20,752 (32.2)	19,447 (31.7)	1305 (42.7)
Child place of birth			
Australia	63,885 (99.3)	60,847 (99.3)	3038 (99.3)
Other country	471 (0.7)	450 (0.7)	21 (0.7)
Child sex			
Male	32,406 (50.4)	30,838 (50.3)	1568 (51.3)
Female	31,950 (49.6)	30,459 (49.7)	1491 (48.7)
Indigenous status			
Nonindigenous	59,689 (92.8)	56,869 (92.8)	2820 (92.2)
Indigenous	4667 (7.2)	4428 (7.2)	239 (7.8)
Language other than English spoken at home			
No	57,148 (88.8)	54,705 (89.3)	2443 (79.9)
Yes	7208 (11.2)	6592 (10.8)	616 (20.1)
Age category of the child at time of AEDC collection			
≥ 4 y to 5 y and 1 mon	11,190 (17.4)	10,578 (17.3)	612 (20.0)
≥ 5 y and 1 mon to 5 y and 10 mon	47,595 (74.0)	45,370 (74.0)	2225 (72.7)
≥ 5 y and 10 mon	5571 (8.6)	5349 (8.7)	222 (7.3)
DV1			
No	49,997 (77.7)	47,709 (77.8)	2288 (74.8)
Yes	14,359 (22.3)	13,588 (22.2)	771 (25.2)
DV2			
No	57,233 (88.9)	54,586 (89.1)	2647 (86.5)
Yes	7123 (11.1)	6711 (10.9)	412 (13.5)

*AEDC* Australian Early Development Census, *DV1/DV2* developmentally vulnerable on one or more AEDC domains/developmentally vulnerable on at least two AEDC domains

### Statistical analysis

**Fig. 1:**
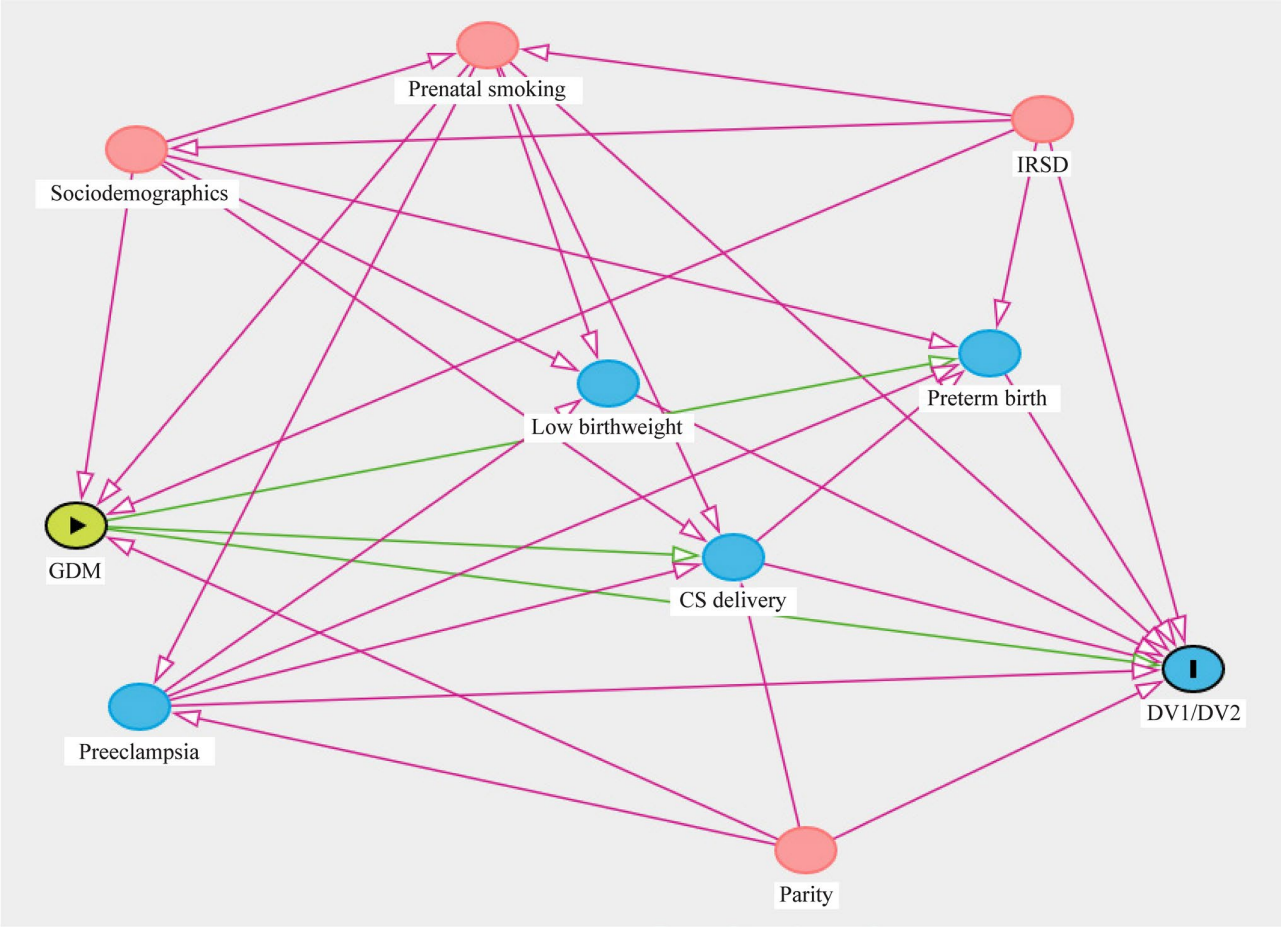
Directed acyclic graph representing the assumed causal mechanism underlying the relationship between gestational diabetes mellitus and childhood developmental vulnerability. *GDM* gestational diabetes mellitus, *AEDC* Australian Early Development Census, *DV1/DV2* developmentally vulnerable on one or more AEDC domains/developmentally vulnerable on at least two AEDC domains, *IRSD* Index of Relative Socioeconomic Disadvantage, *CS* cesarean section

Based on existing evidences from epidemiological studies to represent the potential causal pathway between gestational diabetes mellitus and childhood developmental vulnerability in AEDC domains, we produced a directed acyclic graph (DAG) (Fig. 1). The association between gestational diabetes mellitus and risk of childhood developmental vulnerability in AEDC domains was modelled using generalized linear models (GLM) fitted with a binomial distribution with a log link function to estimate unadjusted relative risks (RRs) with 95% confidence interval (CI) for both outcomes separately using Stata 16.1. Next, causal mediation analyses, based on the counterfactual framework, were undertaken using a parametric-regression-based approach. This approach extends conventional statistical mediation analysis, widely known as the “*Baron and Kenny procedure”,* to further account for the presence of exposure–mediator interactions in the outcome regression model using counterfactual definitions of natural direct (NDE) and indirect (NIE) effects via the mediator [30]. According to this approach [30], two models were estimated: a model for preterm birth (mediator) conditional on gestational diabetes mellitus (exposure) and covariates and a model for DV1/DV2 (outcomes) conditional on exposure, the mediator, and covariates. We fitted both models with maternal age at childbirth, ethnicity/race, marital status, socioeconomic disadvantage quintiles, parity, maternal tobacco smoking during pregnancy, child sex, language other than English spoken at home and indigenous status to estimate the NDE of GDM on childhood developmental vulnerability in AEDC domains, the NIE of GDM on childhood developmental vulnerability in AEDC domains via preterm birth and the marginal total effect (NDE + NIE) with adjusted RR and 95% CI derived using a bootstrap option. Additional sensitivity analyses were conducted to further examine the role of cesarean section delivery in the association between GDM and childhood developmental vulnerability in AEDC domains. We also conducted an additional sensitivity analysis by pooling children who were identified as having “special needs” with those assessed for developmental vulnerability using GLMs. We computed the proportion of the effect mediated by preterm birth [NDE × (NIE − 1)]/(NDE × NIE − 1). We did not include birthweight and preeclampsia in the models, as they are on the causal pathway.

## Results

### Cohort characteristics

Of the 64,356 children, 4.8% were exposed to gestational diabetes mellitus, 15.7% were born to mothers who reported prenatal tobacco smoking, and 6.4% were born preterm. In comparison to women without GDM, women with GDM were more likely to give birth via cesarean section. For the whole cohort, approximately 22% were classified as DV1, and 11% were classified as DV2 (Table 1).

### Natural direct and indirect effects of GDM on early childhood developmental vulnerability in AEDC domains

Findings from an unadjusted model are presented in Table 2. The NDE, NIE, and TE estimates for all mediation models suggest that GDM was associated with DV1 and DV2 (Table 3). The NDE estimates suggested that children exposed to GDM were more likely to be classified as DV1 (RR = 1.15, 95% CI: 1.08–1.24) and DV2 (RR = 1.26, 95% CI: 1.14–1.40) after adjusting for covariates.

**Table 2:** Unadjusted estimates for the association between GDM and DV1 and DV2

Effects	DV1	DV2
	RR (95% CI)	
Gestational diabetes mellitus (MNS)	1.14 (1.07–1.21)	1.23 (1.12–1.35)
Gestational diabetes mellitus (HMDC)	1.16 (1.09–1.24)	1.24 (1.12–1.37)
Gestational diabetes mellitus (MNS + HMDC)	1.17 (1.09–1.26)	1.28 (1.16–1.43)

*GDM* gestational diabetes mellitus, *AEDC* Australian Early Development Census, *DV1/DV2* developmentally vulnerable on one or more AEDC domains/developmentally vulnerable on at least two AEDC domains, *MNS* Midwives Notification System, *HMDC* Hospital Morbidity Data Collection, *RR* relative risk, *CI* confidence interval

**Table 3:** Results of causal mediation analysis adjusted for potential confounders using MNS to define gestational diabetes [relative risk (RR) (95% CI)] (*n* = 64,356)

Effects	DV1	DV2
	RR (95% CI)	
NDE	1.17 (1.09–1.26)	1.28 (1.18–1.40)
NIE	1.01 (1.00–1.01)	1.01 (1.00–1.01)
Marginal total effect	1.18 (1.09–1.27)	1.29 (1.18–1.41)
Proportion mediated by preterm birth = [NDE × (NIE − 1)]/(NDE × NIE − 1)	0.064 (6.40%)	0.044 (4.40%)

Adjusted for maternal age, race, marital status, ethnicity, SES quintiles, parity, prenatal tobacco smoking, sex of child, language, and indigenous status

*AEDC* Australian Early Development Census, *DV1/DV2* developmentally vulnerable on one or more AEDC domains/developmentally vulnerable on at least two AEDC domains, *MNS* Midwives Notification System, *NDE* natural direct effect, *NIE* natural indirect effect, *SES* Index of Relative Socioeconomic Disadvantage *RR* relative risk, *CI* confidence interval

Similarly, the NIE estimates also demonstrated positive associations between gestational diabetes mellitus and DV1 (RR = 1.01, 95% CI: 1.00–1.02) and DV2 (RR = 1.00, 95% CI: 1.00–1.01), suggesting that the positive marginal total effect between GDM and DV1 and DV2 might be partially explained by preterm birth, although minimal in magnitude. About 6% and 4% of the effect of GDM on early childhood developmental vulnerability in AEDC domains was mediated by preterm birth for DV1 and DV2, respectively. Analyses repeated by restricting to pregnant women who were diagnosed with gestational diabetes mellitus at hospital admission revealed similar results (Table 4). Furthermore, additional sensitivity analyses by combining GDM status from the MNS with the hospital diagnosis (HMDC) also suggested similar estimates, indicating a lack of sensitivity of observations to outcome ascertainment (Table 5). The results from additional sensitivity analyses by including cesarean section delivery in the models to further examine its role in the association between GDM and childhood developmental vulnerability in AEDC domains revealed negligible differences in estimates. Similarly, findings from an additional sensitivity analysis by pooling children who were identified as having “special needs” with those assessed for developmental vulnerability suggested negligible variations in estimates (Supplementary Tables 1–6).

**Table 4:** Results of causal mediation analysis adjusted for potential confounders using HMDC to define gestational diabetes [relative risk (RR) (95% CI)] (*n* = 64,356)

Effects	DV1	DV2
	RR (95% CI)	
NDE	1.18 (1.09–1.28)	1.27 (1.14–1.42)
NIE	1.01 (1.00–1.01)	1.01 (1.00–1.01)
Marginal total effect	1.19 (1.09–1.29)	1.28 (1.15–1.43)
Proportion mediated by preterm birth = [NDE × (NIE − 1)]/ (NDE × NIE − 1)	0.062 (6.20%)	0.045 (4.50%)

Adjusted for maternal age, race, marital status, ethnicity, SES quintiles, parity, prenatal tobacco smoking, sex of child, language and indigenous status

*AEDC* Australian Early Development Census, *DV1/DV2* developmentally vulnerable on one or more AEDC domains/developmentally vulnerable on at least two AEDC domains, *HMDC* Hospital Morbidity Data Collection, *NDE* natural direct effect, *NIE* natural indirect effect, *SES* Index of Relative Socioeconomic Disadvantage *RR* relative risk, *CI* confidence interval

**Table 5:** Results of causal mediation analysis adjusted for potential confounders using both MNS and HMDC to define gestational diabetes [relative risk (RR) (95% CI)] (*n* = 64,356)

Effects	DV1	DV2
	RR (95% CI)	
NDE	1.20 (1.10–1.31)	1.34 (1.19–1.50)
NIE	1.01 (1.00–1.01)	1.01 (1.00–1.01)
Marginal total effect	1.21 (1.11–1.31)	1.35 (1.20–1.51)
Proportion mediated by preterm birth = [NDE × (NIE − 1)]/(NDE × NIE − 1)	0.566 (5.66%)	0.038 (3.80%)

Adjusted for maternal age, race, marital status, ethnicity, SES quintiles, parity, prenatal tobacco smoking, sex of child, language and indigenous status

*AEDC* Australian Early Development Census, *DV1/DV2* developmentally vulnerable on one or more AEDC domains/developmentally vulnerable on at least two AEDC domains, *MNS* Midwives Notification System, *HMDC* Hospital Morbidity Data Collection, *NDE* natural direct effect, *NIE* natural indirect effect, *SES* Index of Relative Socioeconomic Disadvantage *RR* relative risk, *CI* confidence interval

## Discussion

In this population-based retrospective cohort study, we estimated the natural direct, indirect, and total effects of GDM on early childhood developmental vulnerability as measured by the AEDC in Western Australia. The results of our study suggest that children born to mothers with GDM were more likely to be classified as DV1 and DV2 at the median age of five years after controlling for potential confounders. These associations did not appear to be mediated by preterm birth or cesarean section delivery.

Consistent with our current findings, previous epidemiological studies have also suggested that GDM was associated with decreased gross and fine motor development [31], lower cognitive scores [11], poorer language development [12, 13], lower IQ and impoverished behavioral and emotional functioning [14], and lower scores of mental and psychomotor development [9], delays in problem-solving ability and personal and social skills [32]. In support of these observations, a population-based prospective study of over 45,000 mothers from Psychiatry Sweden, a linkage of Swedish national registers, observed that gestational diabetes mellitus was associated with an increased risk of neurodevelopmental disorders in their children [33]. However, other studies reported conflicting results [15–17]. For instance, a retrospective population-based study from Australia (*n* = 771) concluded that there is insufficient statistical evidence to claim an association between maternal diabetes mellitus during pregnancy and early childhood developmental vulnerability or risk in gross and fine motor skills, communication skills, and general knowledge AEDC domains in children exposed [15]. Similarly, study data of 772 mother–child pairs from the “Rhea” mother–child cohort in Greece suggested that GDM was not associated with child neurodevelopment at preschool age [34]. It is plausible that those studies may not have been adequately powered to detect differences in childhood developmental outcomes, perhaps due to relatively small sample sizes, variations in the childhood developmental outcomes ascertained or variability in the selection of potential confounders.

Potential pathways explaining the association between GDM and early childhood developmental vulnerability in AEDC domains are still not well explored. The environment in utero relevant to morbidity from GDM is mainly characterized by recurrent acute changes in glucose status and acidemia [35, 36]. Metabolic alterations such as high or fluctuating concentrations of glucose result in higher fetal insulin levels often after the middle of the gestational period [37]. Consistent with findings from in vivo studies [38, 39], GDM is believed to alter normal brain development and structure, thereby increasing the risk of neurocognitive developmental problems in children, including alterations in attention and motor function [40–42] and long-term cognitive ability [43]. Alternatively, rarely occurring metabolic complications induced by the immunologic and metabolic disturbances linked with GDM, such as ketonemia [43], ketoacidosis [44], and glycosuria [45, 46], may also increase the risk of neurodevelopmental disorders in children. While such observations are plausible, our study did not provide direct support for the involvement of any of these mechanisms.

Gestational diabetes mellitus may be linked with inflammation and epigenetic changes in DNA methylation in the placenta and fetal cord blood due to adaptive placental and fetal responses to a greater level of circulating glucose during the early stages of pregnancy [47]. Evidence from in vivo studies also suggest that GDM stimulates microglial activation and chronic inflammatory responses in the brain of the fetus, thereby altering the function of the hippocampus, which is involved in learning and memory [48, 49]. The corresponding overstimulation and improper functioning of the hippocampus may lead to neurodevelopmental disorders later in life [50, 51].

Some of the strengths of our study are that this is a population-based cohort study to estimate the natural direct and indirect effects of gestational diabetes mellitus on early childhood developmental vulnerability in AEDC domains in their children by applying a robust design causal mediation; the use of both clinical and population-based registries to ascertain gestational diabetes mellitus could potentially reduce misclassification bias. The latter approach allowed us to further examine whether the association between GDM and childhood developmental vulnerability in AEDC domains was unique to the individual source population (clinical or population-based). The use of a large sample size and a standardized and validated measurement tool to measure early childhood developmental vulnerability in AEDC domains were additional strengths of our study. Nonetheless, a few caveats should be considered when interpreting the findings of our study. Maternal prenatal substance use, parental mental health problems, prenatal stressful conditions [52, 53], parenting styles [54], and maternal preconception BMI trajectories may contribute to early childhood developmental vulnerability in AEDC domains. However, most of these risk factors are unlikely to be associated with the exposure and, therefore, would not be expected to have biased our results. Nonetheless, as this was an observational study, we cannot rule out the possibility of residual confounding. Moreover, the AEDC results did not include children who were identified as having “special needs” due to a medical diagnosis or diagnosis of physical or chronic medical conditions or intellectual disability. Therefore, the findings of this study may not be generalized to this population.

In conclusion, children exposed to gestational diabetes mellitus were more likely to be developmentally vulnerable in one or more AEDC domains. The biological mechanism for these associations is not well explained by mediation through preterm birth.

### Supplementary Information

Below is the link to the electronic supplementary material.

Supplementary file1 (DOCX 17 KB)

## Data Availability

All data generated or analyzed during this study were included in this manuscript and are attached as supporting information.
