# Supplementary material for: Influence of preterm birth on the association between gestational diabetes mellitus and childhood developmental vulnerability: a causal mediation analysis
Source: World J Pediatr. 2023 Jul 31;20(1):54–63. doi: 10.1007/s12519-023-00741-7 (PMC10827844; doi:10.1007/s12519-023-00741-7)
Supplement: Supplementary file 1 — Supplementary file1 (DOCX 17 KB) [file 12519_2023_741_MOESM1_ESM.docx]

**Influence of preterm birth on the association between gestational diabetes mellitus and childhood developmental vulnerability: a causal mediation analysis.**

**Supplementary files**

**Supplementary file 1.** Results of Causal Mediation Analysis Adjusted for Potential Confounders using both MNS and HMDC to define gestational diabetes [Relative risk (RR) (95% CI] (n=64,356) by including caesarean section delivery in the model.

| Effect | DV1 | DV2 |
| --- | --- | --- |
|  | RR (95% CI) | |
| Natural direct effect (NDE) | 1.16 (1.08-1.26) | 1.27 (1.15-1.41) |
| Natural Indirect Effect (NIE) | 1.01 (1.00-1.01) | 1.01 (1.00-1.01) |
| Marginal total effect | 1.17 (1.08-1.26) | 1.28 (1.16-1.42) |
| Proportion mediated by preterm birth =(NDE*(NIE-1)) / (NDE*NIE -1) | 0.067 (6.70%) | 0.045 (4.50%) |

Adjusted for maternal age, race, marital status, ethnicity, Index of Relative Socioeconomic Disadvantage quintiles, parity, prenatal tobacco smoking, sex of child, language, indigenous status and caesarean section delivery.

**Supplementary file 2.** Results of Causal Mediation Analysis Adjusted for Potential Confounders using HMDC to define gestational diabetes [Relative risk (RR) (95% CI] (n=64,356) by including caesarean section delivery in the model.

| Effect | DV1 | DV2 |
| --- | --- | --- |
|  | RR (95% CI) | |
| Natural direct effect (NDE) | 1.18 (1.09-1.28) | 1.26 (1.13-1.41) |
| Natural Indirect Effect (NIE) | 1.01 (1.00-1.01) | 1.01 (1.00-1.01) |
| Marginal total effect | 1.19 (1.08-1.29) | 1.27 (1.14-1.42) |
| Proportion mediated by preterm birth =(NDE*(NIE-1)) / (NDE*NIE -1) | 0.062 (6.20%) | 0.046 (4.60%) |

Adjusted for maternal age, race, marital status, ethnicity, Index of Relative Socioeconomic Disadvantage quintiles, parity, prenatal tobacco smoking, sex of child, language, indigenous status and caesarean section delivery.

**Supplementary file3.** Results of Causal Mediation Analysis Adjusted for Potential Confounders using both MNS and HMDC to define gestational diabetes [Relative risk (RR) (95% CI] (n=64,356) by including caesarean section delivery in the model.

| Effect | DV1 | DV2 |
| --- | --- | --- |
|  | RR (95% CI) | |
| Natural direct effect (NDE) | 1.19 (1.11-1.29) | 1.33 (1.19-1.49) |
| Natural Indirect Effect (NIE) | 1.01 (1.00-1.01) | 1.01 (1.00-1.01) |
| Marginal total effect | 1.20 (1.12-1.30) | 1.34 (1.19-1.50) |
| Proportion mediated by preterm birth =(NDE*(NIE-1)) / (NDE*NIE -1) | 0.059 (5.90%) | 0.038 (3.80%) |

Adjusted for maternal age, race, marital status, ethnicity, Index of Relative Socioeconomic Disadvantage, parity, prenatal tobacco smoking, sex of child, language, indigenous status and caesarean section delivery.

**Supplementary file 4**. Results of Causal Mediation Analysis Adjusted for Potential Confounders using HMDC to define gestational diabetes [Relative risk (RR) (95% CI] (n=64,356)

| Effect | DV1 | DV2 |
| --- | --- | --- |
|  | RR (95% CI) | |
| Natural direct effect (NDE) | 1.17 (1.10-1.26) | 1.26 (1.15-1.38) |
| Natural Indirect Effect (NIE) | 1.01 (1.01-1.02) | 1.01 (1.00-1.01) |
| Marginal total effect | 1.18 (1.11-1.28) | 1.27 (1.16-1.39) |
| Proportion mediated by preterm birth =(NDE*(NIE-1)) / (NDE*NIE -1) | 0.064 (6.4%) | 0.046 (4.6%) |

Adjusted for maternal age, race, marital status, ethnicity, Index of Relative Socioeconomic Disadvantage quintiles, parity, tobacco smoke, child age category, sex of child, language, indigenous status, baby place of birth and caesarean section delivery.

**Supplementary file 5.** Results of Causal Mediation Analysis Adjusted for Potential Confounders using both MNS and HMDC to define gestational diabetes [Relative risk (RR) (95% CI] (n=64,356)

| Effect | DV1 | DV2 |
| --- | --- | --- |
|  | RR (95% CI) | |
| Natural direct effect (NDE) | 1.19 (1.10-1.30) | 1.32 (1.18-1.49) |
| Natural Indirect Effect (NIE) | 1.01 (1.00-1.02) | 1.01 (1.00-1.01) |
| Marginal total effect | 1.20 (1.10-1.31) | 1.33 (1.19-1.50) |
| Proportion mediated by preterm birth =(NDE*(NIE-1)) / (NDE*NIE -1) | 0.058 (≈6%) | 0.039 (≈4%) |

Adjusted for maternal age, race, marital status, ethnicity, Index of Relative Socioeconomic Disadvantage quintiles, parity, tobacco smoke, child age category, sex of child, language, indigenous status, baby place of birth and caesarean section delivery.

**Supplementary file 6.** Results of Causal Mediation Analysis Adjusted for Potential Confounders using both MNS and HMDC to define gestational diabetes including children who were categorised under “special needs” [Relative risk (RR) (95% CI] (n=67,079)

| Effect | DV1 | DV2 |
| --- | --- | --- |
|  | RR (95% CI) | |
| Natural direct effect (NDE) | 1.17 (1.10-1.26) | 1.26 (1.15-1.38) |
| Natural Indirect Effect (NIE) | 1.01 (1.01-1.02) | 1.01 (1.00-1.01) |
| Marginal total effect | 1.18 (1.11-1.28) | 1.27 (1.16-1.39) |

Adjusted for maternal age, race, marital status, ethnicity, Index of Relative Socioeconomic Disadvantage quintiles, parity, tobacco smoke, child age category, sex of child, language, indigenous status, baby place of birth and caesarean section delivery.
